# Hedgehog pathway inhibition causes primary follicle atresia and decreases female germline stem cell proliferation capacity or stemness

**DOI:** 10.1186/s13287-019-1299-5

**Published:** 2019-07-05

**Authors:** Yu Jiang, Dantian Zhu, Wenfeng Liu, Qiushi Qin, Zhi Fang, Zezheng Pan

**Affiliations:** 1 Faculty of Basic Medical Science, Jiangxi Medical College, Nanchang University, Nanchang, 330006 China; 2 Medical College, Nanchang University, Nanchang, Jiangxi Province China

**Keywords:** Hedgehog signaling pathway, Female germline stem cell, Follicular number, Proliferation, Stemness

## Abstract

**Background:**

Follicle depletion is one of the causes of premature ovarian failure (POF) and primary ovarian insufficiency (POI). Hence, maintenance of a certain number of female germline stem cells (FGSCs) is optimal to produce oocytes and replenish the primordial follicle pool. The mechanism that regulates proliferation or stemness of FGSCs could contribute to restoring ovarian function, but it remains uncharacterized in postnatal mammalian ovaries. This study aims to investigate the mechanism by which inhibiting the activity of the hedgehog (Hh) signaling pathway regulates follicle development and FGSC proliferation.

**Methods and results:**

To understand the role of the Hh pathway in ovarian aging, we measured Hh signaling activity at different reproductive ages and the correlation between them in physiological and pathological mice. Furthermore, we evaluated the follicle number and development and the changes in FGSC proliferation or stemness after blocking the Hh pathway in vitro and in vivo. In addition, we aimed to explain one of the mechanisms for the FGSC phenotype changes induced by treatment with the Hh pathway-specific inhibitor GANT61 via oxidative stress and apoptosis. The results show that the activity of Hh signaling is decreased in the ovaries in physiological aging and POF models, which is consistent with the trend of expression levels of the germline stem cell markers Mvh and Oct4. In vitro, blocking the Hh pathway causes follicular developmental disorders and depletes ovarian germ cells and FGSCs after treating ovaries with GANT61. The proliferation or stemness of cultured primary FGSCs is reduced when Hh activity is blocked. Our results show that the antioxidative enzyme level and the ratio of Bcl-2/Bax decrease, the expression level of caspase 3 increases, the mitochondrial membrane potential is abnormal, and ROS accumulate in this system.

**Conclusions:**

We observed that the inhibition of the Hh signaling pathway with GANT61 could reduce primordial follicle number and decrease FGSC reproductive capacity or stemness through oxidative damage and apoptosis.

**Electronic supplementary material:**

The online version of this article (10.1186/s13287-019-1299-5) contains supplementary material, which is available to authorized users.

## Background

In mammals, female germline stem cells (FGSCs), also known as ovarian germline stem cells (OGSCs), have been proven to be present in postnatal ovarian surface epithelium (OSE) [1–3]. The major functions of FGSCs are to differentiate into oocytes and replenish the primordial follicle pool. Zou et al. [4, 5] discovered that a dysfunctional ovary can restore the follicles at all stages and produce healthy offspring after transplantation of FGSCs into infertile mice. Therefore, the proliferation capacity of FGSCs contributes to maintaining folliculogenesis and extending the reproductive cycle [6]. Depletion of FGSCs causes a decrease in oocyte number and ovary dysfunction [7]. Exploring the potential mechanism of the regulation of FGSC proliferation or stemness is beneficial to understanding how normal egg and ovary function is maintained. As a canonical signaling pathway, the hedgehog (Hh) pathway plays a key role in the regulation of stem cell proliferation and embryonic development, as well as cellular communication [8–10]. Studies showed that mice were subjected to serious damage in folliculogenesis when a mutation was introduced in the Hh pathway [11]. In addition, the Hh pathway is also involved in the development of germ cells in the gonads by affecting the local microenvironment of stem cells [12, 13]. Although many studies have reported that the Hh pathway is relevant to germ cells [14], very little is known about the role of Hh signaling in the regulation of FGSC proliferation and stemness. In view of the above, it is meaningful to explore the function of the Hh pathway in FGSC proliferation and follicular development, which could lead to a new mechanism or method for premature ovarian failure (POF) therapy.

Mouse Vasa homolog (Mvh) is an ATP-dependent RNA helicase and is expressed in all stages of germ cells. Germ cells appear to have arrested differentiation and increased apoptosis in Mvh-mutant mice. As a crucial gene in germ cell development, Mvh is known as the germline cell-specific marker gene [15–17]. Oct4/Pou5f1 belongs to the POU domain family of transcription factors and plays a key role in embryonic development and stem cell pluripotency; it is considered a marker of stem cells and germ cells because of its expression in these cells [18]. Hence, in this study, the specific molecular markers Mvh and Oct4 were used to evaluate the proliferative capacity or stemness of FGSCs [19, 20]. GLI family zinc finger 1 (Gli1), the positive transcription factor of the Hh signaling pathway, is activated by the Hh signal transduction cascade and promotes stem cell proliferation [21]. Membrane receptor Ptch1, a key component of the Hh pathway, and Cyclin D1, one of the Hh pathway target proteins, are both upregulated when the Hh pathway is activated [22, 23]. Here, Gli1, Ptch1, and Cyclin D1 were used to assess the Hh signaling pathway activity. GANT61, a specific Hh pathway inhibitor, was used to observe the phenotype change of FGSCs and ovary tissue in vivo and in vitro [24, 25]. The superoxide dismutase 2 (SOD2) protein binds superoxide byproducts and converts them to hydrogen peroxide and diatomic oxygen [26]. Antioxidase glutathione peroxidase (GPx) protects cells against oxidative damage by catalyzing the reduction of organic hydroperoxides and hydrogen peroxide [27]. Glutathione (GSH) is an important tripeptide that protects the cell against the noxious effects of oxidative material [28]. Malondialdehyde (MDA), a representative lipid peroxide, is generated in cells when oxygen radical levels increase [29]. The activity of SOD2 and GPx, as well as the concentration of GSH and MDA, was used to measure the oxidative stress level in this study [30, 31]. Bcl-2 is a mitochondrial membrane protein that blocks the apoptotic cell death [32]. The protein Bax forms a heterodimer with Bcl-2 and functions as an apoptotic activator [33]. Hence, we selected the anti-apoptotic molecule Bcl-2; pro-apoptotic factors Bax, P16, and P21; and apoptotic marker molecule caspase 3 to evaluate the occurrence of apoptosis [34–36].

There are many kinds of cells in the ovary, such as oocytes, FGSCs, granular cells, and some matrix cells. To avoid interference by granular cells and matrix cells, we chose the OSE, which is mostly composed of germline cells, to detect Hh pathway activity. First, we measured the expression level of Hh pathway members at different reproductive ages in mouse OSE. Furthermore, we determined if colocalization exists between germline stem cell markers and Hh pathway molecules. We developed a POF model by intraperitoneal injection of cyclophosphamide in adult mice to observe changes in Hh signaling activity under pathological conditions. Next, we isolated neonatal mouse ovaries and cultured them with medium containing GANT61, and we counted the number of primordial follicles and FGSCs at different dosages. In addition, a modified two-step enzyme digestion was used to isolate FGSCs, and the primary cultured cells were subcultured and treated with GANT61 in vitro to observe the changes in cellular proliferation or stemness. Finally, we attempted to confirm whether altering the microenvironment by blocking the Hh signaling activity caused phenotypic changes in FGSCs via the induction of oxidative stress injury and apoptosis pathways. Our study investigated whether the Hh signaling pathway is involved in the proliferation of FGSCs and follicle development.

## Materials and methods

### Animals

Clean-grade Kunming (KM) mice were obtained from the Jiangxi University of Traditional Chinese Medicine and were used in all experiments. The mice had free access to food and water. All procedures involving mice were approved by the Animal and Ethics Committee of Nanchang University.

### Ovary extract and culture

Under sterile conditions, mice of different ages were sacrificed and the ovaries were extracted. The ovarian appendage was carefully cleared, and the intact ovaries were rinsed in precooled D-Hanks solution. For ovary culture in vitro, ovaries were placed on a gelatin sponge surface in Waymouth medium (Sigma, USA) containing 10% (*v*/*v*) FBS (Gibco, USA), 0.23 mM sodium pyruvate, and 100× penicillin and streptomycin (P/S) (Solarbio, China).

### POF model preparation

Approximately 4–6-week-old KM mice were used to construct the POF model by intraperitoneal injection of busulfan (30 mg/kg, Sigma, USA) and cyclophosphamide (120 mg/kg, Sigma, USA). The control group was given normal saline in the same manner. Three to 4 weeks after injection, there were no normal follicles and no offspring, which represented successful establishment of the model.

### FGSC primary isolation and culture

Twelve to 16 ovaries from suckling mice (3–5 days old) were collected and cleaned in precooled D-Hanks solution, and then primary FGSCs were isolated via the modified two-step enzymatic digestion method. In brief, ovaries were cut into 2–4 pieces in D-Hanks solution without calcium and magnesium and transferred into collagenase IV (1 mg/ml, Sigma) solution. After digesting the section for 13 min at 37 °C, the solution was spun at 300 g for 5 min, then the pellets were resuspended in 0.05% trypsin containing EDTA (1 mM, Sigma) and incubated 3 min at 37 °C after washing with D-Hanks solution 2 times. Finally, trypsin digestion was stopped with serum, and the primary cells were cultured with FGSC-specific medium containing minimum essential medium α (MEM-α), 10% FBS, 1 mM sodium pyruvate, 1 mM nonessential amino acids, 2 mM l-glutamine, 0.1 mM β-mercaptoethanol (Sigma), 20 ng/ml LIF (Sigma), 10 ng/ml mEGF (Sigma), 40 ng/ml GDNF (Sigma), 1 ng/ml bFGF (Sigma), and 100× penicillin and streptomycin (P/S) (Solarbio, China). The medium was changed every other day, and cells were subcultured at approximately 80% cellular density.

### Immunoblotting, immunohistochemistry, and immunofluorescence

Immunoblotting (IB), immunohistochemistry (IHC), and immunofluorescence (IF) were performed as described previously [25]. Images were acquired using a NIKON Eclipse 80i microscope. The primary antibodies used in this study include Mvh (Abcam, ab27591), Oct4 (Abcam ab18976), Gli1 (Affinity, DF7523), Ptch1 (Affinity, AF5202), Cyclin D1 (Abcam, ab16663), and GAPDH (Abcam, ab181602). The EdU kit was purchased from Keygen BioTECH (KGA337-1000). All HRP- and fluorophore-conjugated secondary antibodies were obtained from Affinity Biosciences.

### Quantitative real-time PCR

Total RNA from each sample was extracted with Tri Reagent (Life Technologies, USA) and then reverse transcribed using the PrimeScript RT reagent Kit with a gDNA Eraser (TaKaRa, Japan). Amplification was performed using the iTaq Universal SYBR Green Supermix Kit (BIO-RAD) with 40 cycles of 95 °C for 15 s and 60 °C for 1 min on a StepOnePlus Real-Time PCR System. The relative expression level of each transcript was normalized to murine GAPDH by using the 2^(ΔΔCt) method. Table 1 contains the list of primers used in this study.

**Table 1:** Primer sequences used for quantitative real-time PCR

Genes	Primer Sequence(5′-3′)	Gene number
Gli1	Forward: CCCAATACATGCTGGTGGTG Reverse: GCAACCTTCTTGCTCACACA	NM_010296.2
PTCH1	Forward: TGTGCGCTGTCTTCCTTCTG Reverse: ACGGCACTGAGCTTGATTC	NM_001328514.1
CyclinD1	Forward: GATGAGGAAGAGTTGCTAGAAGAG Reverse: TCGTCAGCCAATCGGTAGTAG	NM_007631.2
Mvh	Forward: GTGTATTATTGTAGCACCAACTCG Reverse: CACCCTTGTACTATCTGTCGAACT	NM_001145885.1
Oct-4	Forward: AGCTGCTGAAGCAGAAGAGG Reverse: GGTTCTCATTGTTGTCGGCT	NM_013633.3
Stella	Forward: CCCAATGAAGGACCCTGAAAC Reverse: AATGGCTCACTGTCCCGTTCA	NM_139218.1
Fragilis	Forward: CTGGTCCCTGTTCAATACACTCTT Reverse: CAGTCACATCACCCACCATCTT	NM_025378.2
Nanog	Forward: TCTCCTCGCCCTTCCTCTGA Reverse: TCCGCATCTTCTGCTTCCTG	NM_001289828.1
Bcl-2	Forward: GAACTGGGGGAGGATTGTGG Reverse: GCATGCTGGGGCCATATAGT	NM_009741.5
Bax	Forward: GAACCATCATGGGCTGGACA Reverse: AGCCACCCTGGTCTTGGAT	NM_007527.3
P16	Forward: AAGGCTTTGAGAGCCCATCT Reverse: CACTTCTGTGAACGGTGTCC	NM_001040654.1
P21	Forward: TAGAGAGCTGGATGCCACTG Reverse: CCTGGCATAGCCAGTGTAGA	NM_001111099.2
GAPDH	Forward: CGTGCCGCCTGGAGAAACCTG Reverse: AGAGTGGGAGTTGCTGTTGAAGTCG	NM_001289726.1

### Alkaline phosphatase staining and activity assay

Cells were fixed in 4% formalin for 10 min and washed 3 times with PSB. Alkaline phosphatase (ALP) staining and activity assays were performed with a staining kit (Solarbio, G1480) and an activity assay kit (Solarbio, BC2140) according to the manufacturer’s instructions.

### Cell proliferation assay and colony formation assay

FGSCs (2000 cells per well) were plated in 96-well plates, treated for 48 h with GANT61 (HY-13901, MedChemExpress, USA), and then measured using a Cell Counting Kit (CCK-8, Transgen BioTECH, China). In addition, 2000 FGSCs were seeded in 6 cm plates and cultured for 7 days under GANT61 treatment. Crystal violet was used to stain and count the number of cellular colonies.

### Mitochondrial membrane potential measurement

The JC-10 apoptosis detection kit (KeyGen BioTECH, China) was used to detect mitochondrial membrane potential. FGSCs were washed three times in sterile PBS, and JC-10 working solution was added to each well and incubated for 30 min under 5% CO_2_ and 37 °C conditions. Hoechst 33342 (Solarbio B8040) was used to stain the nuclei. After incubation, the FGSCs were washed with PBS three times and observed under a NIKON Eclipse 80i fluorescence microscope. The green fluorescent channel image (FL1) and the red fluorescent channel image (FL2) were analyzed by ImageJ software, and the ratio of FL2 to FL1 was calculated to reflect the mitochondrial membrane potential.

### Detection of intracellular reactive oxygen species levels

Dihydrorhodamine (DHR, KeyGen BioTECH, China) was used to measure the total intracellular ROS levels following the kit instructions. In short, 10 μM of DHR working solution was added to the wells with FGSCs and incubated for 1 h in the dark. Hoechst 33342 was used to stain the nuclei. A NIKON Eclipse 80i fluorescence microscope was used to observe the staining intensity, and ImageJ software was used to analyze the fluorescence images and perform statistical analysis.

### Statistical analysis

Two-tailed Student’s *t* test to compare two groups or one-way ANOVA followed by planned comparisons to compare multiple groups were performed with GraphPad Prism 7 software. A *p* value < 0.05 was considered statistically significant. All data are presented as the mean ± SD error from at least three independent experiments.

## Results

### Hh signaling pathway activity decreases with ovarian aging

**Fig. 1:**
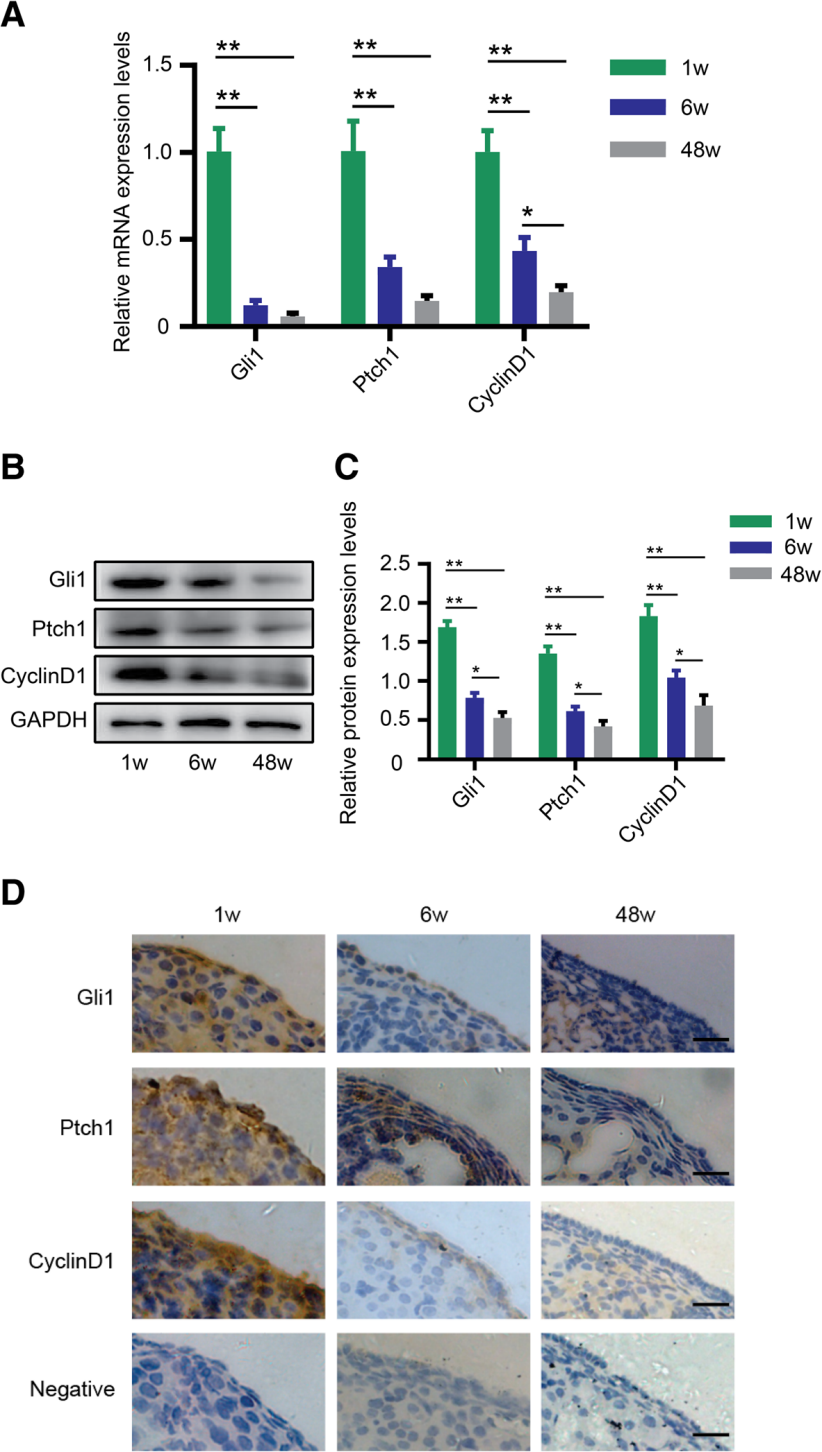
Hh signaling pathway activity decreases with ovarian aging. **a** mRNA expression levels of Gli1, Ptch1, and Cyclin D1 in three mice of reproductive age. **b**, **c** Protein expression levels. **d** IHC detection of key members of the Hh pathway, Gli1, Ptch1, and Cyclin D1, in the OSE. Bar is 10 μm. *p < 0.05 and **p < 0.01

To measure Hh signaling activity at different ages, we chose 1-week-old, 6-week-old, and 48-week-old normal mouse ovaries to represent infancy, adulthood, and senescence reproductive phases, respectively. RT-PCR analysis of OSE tissue showed that Gli1, Ptch1, and Cyclin D1 expression was the highest at 1 week, decreased significantly at 6 weeks, and declined dramatically at 48 weeks (Fig. 1a, *p <* 0.01). The western blotting results indicated a similar trend corresponding to the mRNA expression levels (Fig. 1b, c, *p <* 0.01 or *p <* 0.05). In addition, IHC experiment results demonstrated the darkest Gli1 staining at 3 days, an intermediate staining at 2 months, and the lightest staining at 12 months of age. A similar staining trend was observed for Ptch1 and Cyclin D1 (Fig. 1d).

### Ovary aging closely correlates with a decrease in Hh pathway activity

**Fig. 2:**
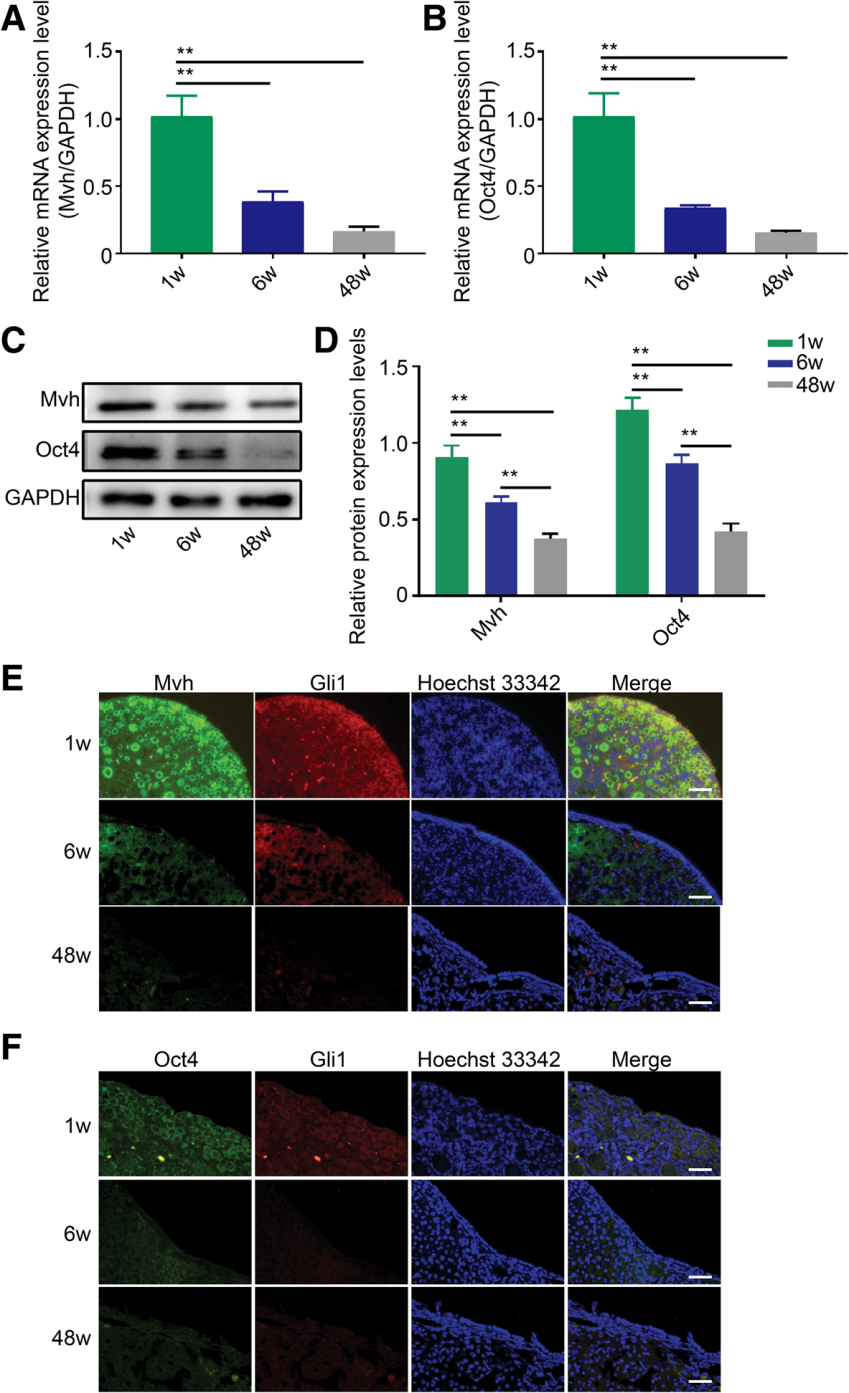
Ovarian aging has a similar trend as the decrease in Hh activity. **a** mRNA levels of Mvh decrease gradually along with ovarian aging. **b** Oct4 mRNA levels have the same trend as Mvh levels. **c**, **d** Protein levels of Mvh and Oct4 at three reproductive age points. **e**, **f** Dual-IF showed the coexpression of Mvh and Gli1, Oct4, and Gli1 in OSE. Bar is 20 μm. *p < 0.05 and **p < 0.01

First, we measured the reproductive activity of normal mice at different ages. As shown in Fig. 2a, c, and d, the specific germline cell marker Mvh was expressed at the highest level at 1 week of age, at a relatively high level at 6 weeks of age, and evidently decreased at 48 weeks of age (*p* < 0.01). Expression levels of the stem cell marker Oct4 also continuously decreased along with ovarian aging (Fig. 2b–d, *p* < 0.01). To investigate the correlation between Hh signaling pathway activity and ovarian aging, we measured the relative coexpression of molecules in the OSE by dual-IF. As shown in Fig. 2e, both Mvh and Gli1 had the strongest fluorescence at 1 week of age, and an obvious drop and a dramatic decrease in fluorescence were observed at 6 weeks and 48 weeks of age, respectively. This result demonstrated that germline cells abound in infant ovaries and are almost exhausted in senescent ovaries. Dual staining of Oct4 and Gli1 exhibited similar results (Fig. 2f), suggesting that the number of FGSCs or germline cells decreases with age.

### The pathological ovary exhibits a decline in Hh pathway activity

**Fig. 3:**
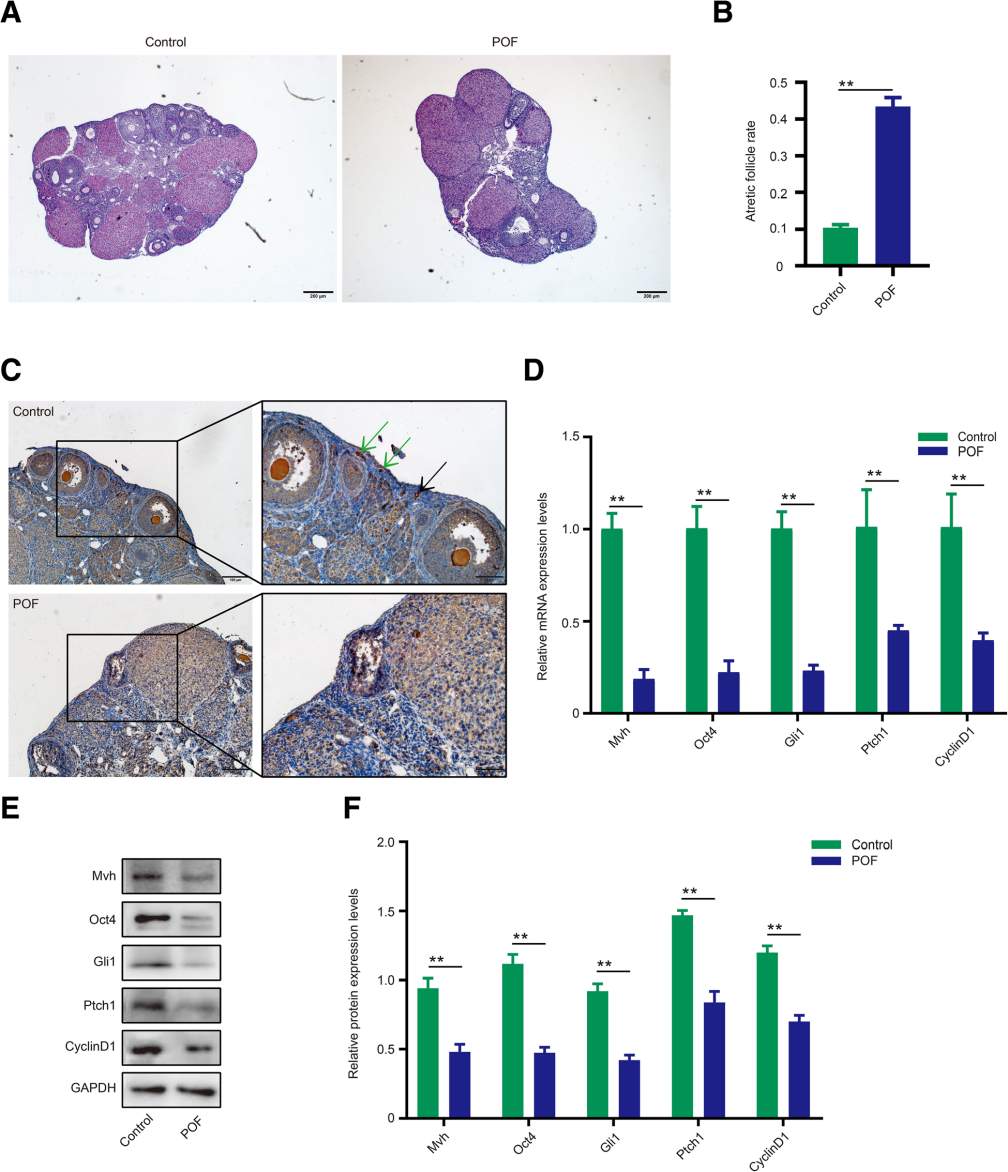
Pathological ovaries exhibited a decline in Hh pathway activity. **a** HE staining of a normal ovary and POF ovary. Bar is 200 μm. **b** The ratio of atretic follicles of normal and POF ovaries. **c** IHC shows that the Mvh-positive follicles and FGSCs dramatically disappeared in the POF model; green arrows are FGSCs, and black arrow represents primordial follicles, the scale is 100 μm. **d**–**f** mRNA and protein expression levels of Mvh, Oct4, Gli1, Ptch1, and Cyclin D1. *p < 0.05 and **p < 0.01

To further assess the relationship between germline cells and Hh signaling, 4–6-week-old mice were used to generate pathological ovaries via treatment with chemotherapy drugs. Compared with the normal group, the POF ovaries exhibited normal follicle descent and atretic follicle increase (Fig. 3a, b, *p* < 0.01), and both primary follicles and FGSCs almost disappeared in the pathological OSE (Fig. 3c). Next, we measured the Hh signaling activity. Interestingly, Gli1, Ptch1, and Cyclin D1 mRNA expression levels decreased to various degrees, particularly Gli1 levels at over 70%, and protein levels also showed similar results (Fig. 3d–f, *p* < 0.01). The decrease in Mvh and Oct4 expression levels also verified the damage to the ovaries in the POF model (Fig. 3d–f, *p* < 0.01). Together, our results indicated a decline in normal follicles and Hh pathway activity in the pathological ovaries.

### Inhibition of Hh signaling decreased the follicle number and FGSC proliferation or stemness in the ovary

**Fig. 4:**
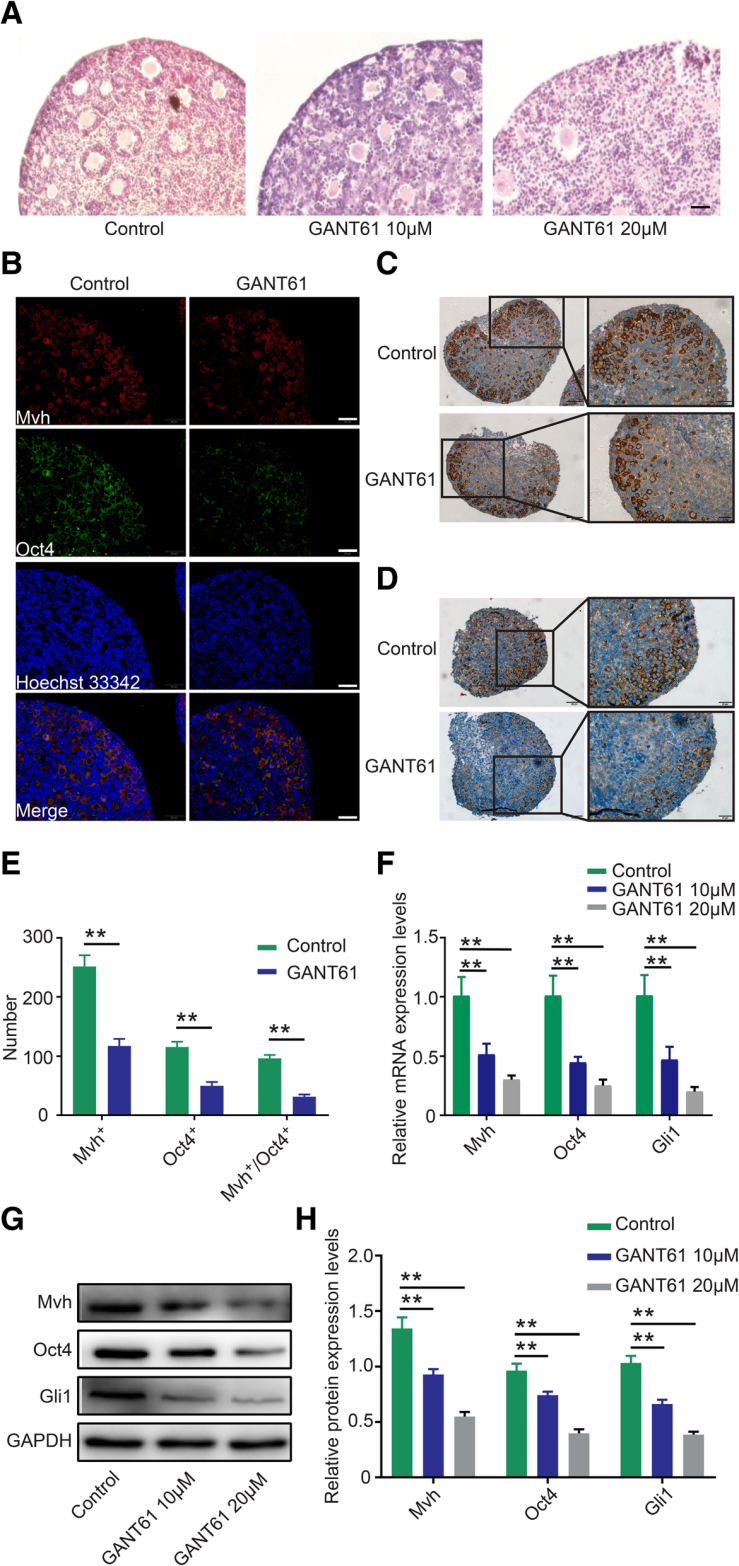
Inhibition of Hh signaling decreased the follicle number and FGSC proliferation. **a** HE staining shows a dramatic drop in primary follicle number after treatment with GANT61, the scale is 20 μm. **b** Dual-IF detects coexpression of Oct4 and Mvh, bar is 20 μm. **c**, **d** IHC of Mvh and Oct4, individually, the scale is 50 μm. **e** The number of cells positive for Mvh and Oct4 and double positive for Mvh/Oct4. **f** mRNA expression levels of Mvh and Oct4. **g**, **h** Protein expression of Mvh and Oct4. *p < 0.05 and **p < 0.01

To further define the role of Hh signaling in ovarian tissue, we extracted 3–5-day-old ovaries (*n* = 6) and cultured them for 48 h in vitro with the Hh pathway-specific inhibitor GANT61. The HE staining results showed that primary follicles decreased over 50% in the 10 μM group, and follicles had almost disappeared in the 20 μM group (Fig. 4a), indicative of severe loss of follicle number after blocking the Hh pathway. FGSCs were mainly located in the OSE, and we attempted to count the number change of FGSCs. Dual-IF staining revealed that the number of cells in the GANT61 group coexpressing both Mvh and Oct4 decreased by more than half (from approximately 90 to 40) compared with the control group (Fig. 4b, e, *p* < 0.01). The IHC results for Mvh indicated that the germline cell numbers were approximately 280 in the normal ovary and approximately 110 in the GNAT61 group (Fig. 4c, e, *p* < 0.01), indicating that the follicle stock was seriously damaged. Additionally, 113 cells in the control group expressed Oct4, corresponding to 51 cells in the GANT61 ovary (Fig. 4d, e, *p* < 0.01), suggesting that FGSCs lost their self-renewal capacity in the treated tissue. Next, we measured the expression levels of Mvh and Oct4 in GANT61-treated ovaries. As shown in Fig. 4f–h, a visible reduction in Mvh and Oct4 levels was observed in the 10 μM group, and a significant decrease was observed in the 20 μM group (*p* < 0.01). Altogether, these results demonstrated that inhibition of the Hh pathway caused primary follicular atresia and decreased FGSC proliferation in ovaries.

### Attenuating Hh signaling decreased FGSC proliferation in vitro

**Fig. 5:**
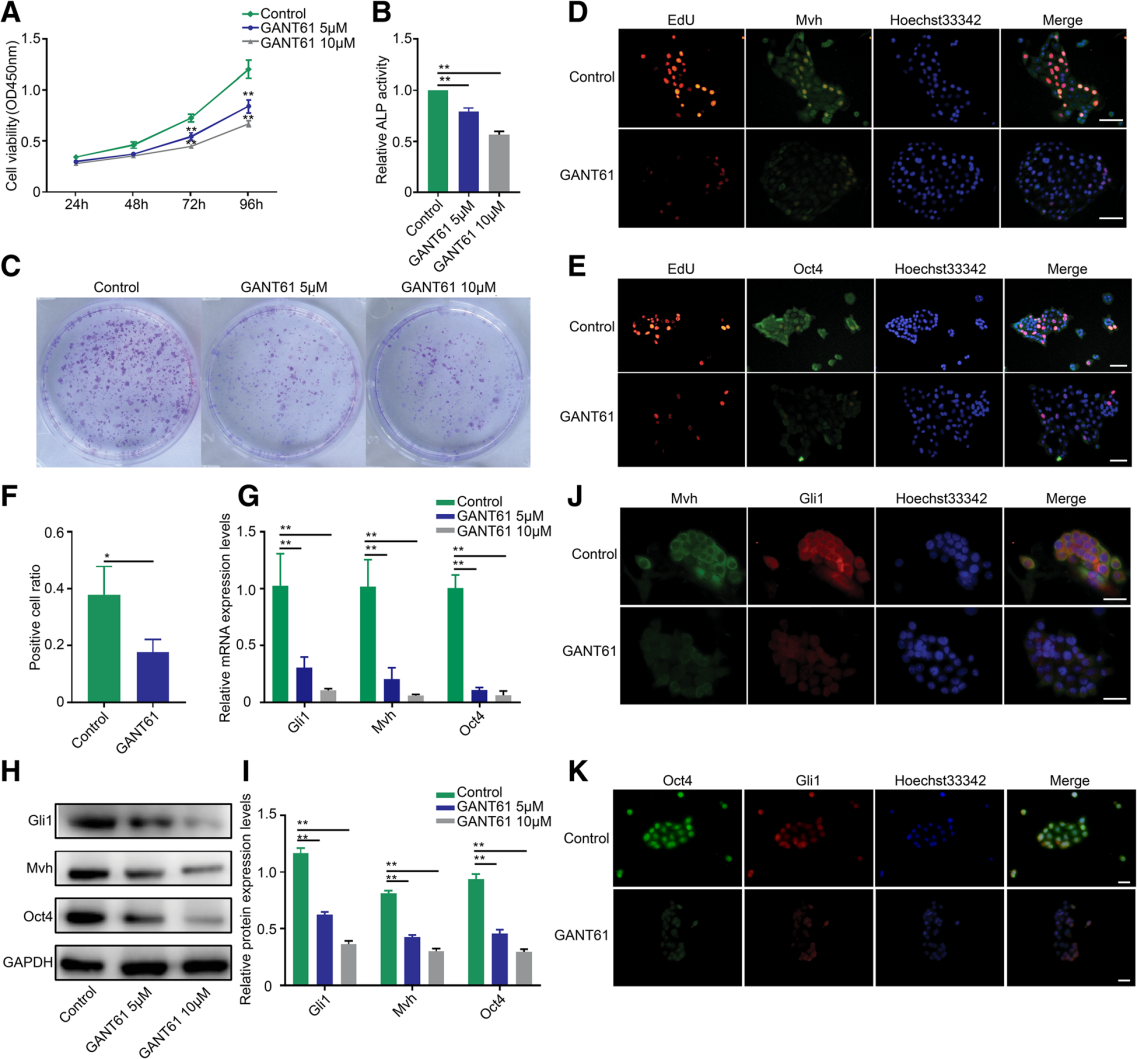
Attenuating Hh signaling decreased FGSC proliferation in vitro. **a** CCK-8 assay was used to measure the proliferation activity of FGSCs cells after GANT61 treatment. **b** Change in ALP activity of FGSCs after different GANT61 dosages. **c** FGSC colony formation assays. **d**, **e** EdU staining combined with IF of Mvh and Oct4 comparing the fluorescence intensity between the control and GANT61 10 μM groups, scale is 20 μm. **f** FGSC proliferation activity was evaluated by EdU-positive cell rate. **g** mRNA expression levels of Gli1, Mvh, and Oct4 after GANT61 treatment. **h**, **i** Protein expression levels of Gli1, Mvh, and Oct4. **j**, **k** Dual-IF was used to detect the coexpression of Oct4 and Mvh in the control and GANT61 10 μM groups, the scale is 20 μm. *p < 0.05 and **p < 0.01

In addition to looking at the animal level, we explored whether the Hh pathway also regulates FGSC growth or viability at the cellular level. To this end, primary FGSCs were cultured and treated with different doses of GANT61. First, CCK-8 assays were used to measure the proliferation of FGSCs. As expected, the growth of FGSCs decreased in response to treatment in a concentration-dependent manner (Fig. 5a, *p* < 0.01). Furthermore, ALP analysis showed that ALP activity decreased by approximately 20% in the 5 μM group and by almost 50% in the 10 μM group after 48 h of treatment (Fig. 5b, *p* < 0.01). In addition, colony formation assays showed that colony numbers were dramatically decreased in both the 5 μM and 10 μM groups, but there was no visible distinction between the 5 μM and 10 μM groups (Fig. 5c). In addition, as shown in Fig. 5d and f, the EdU-positive cells in the 10 μM group were reduced by over 50% compared with the control group (*p* < 0.05), and Mvh fluorescence decreased correspondingly, suggesting an inhibition of cell growth in FGSCs. The same phenomenon was observed in Oct4/EdU detection experiments (Fig. 5e).

To evaluate the overall effects of the Hh pathway on FGSCs, we analyzed RNA and protein levels using cells co-cultured with GANT61 for 48 h. In agreement with the in vivo data, both Mvh and Oct4 levels were downregulated in the GANT61-treated group (Fig. 5g–i, *p* < 0.01). In addition, the fluorescence of Mvh/Gli almost disappeared in the 10 μM group, and the same phenomenon was observed in the Oct/Gli group (Fig. 5j, k).

### Suppressing Hh pathway activity reduced the antioxidative stress ability of FGSCs and prompted apoptosis

**Fig. 6:**
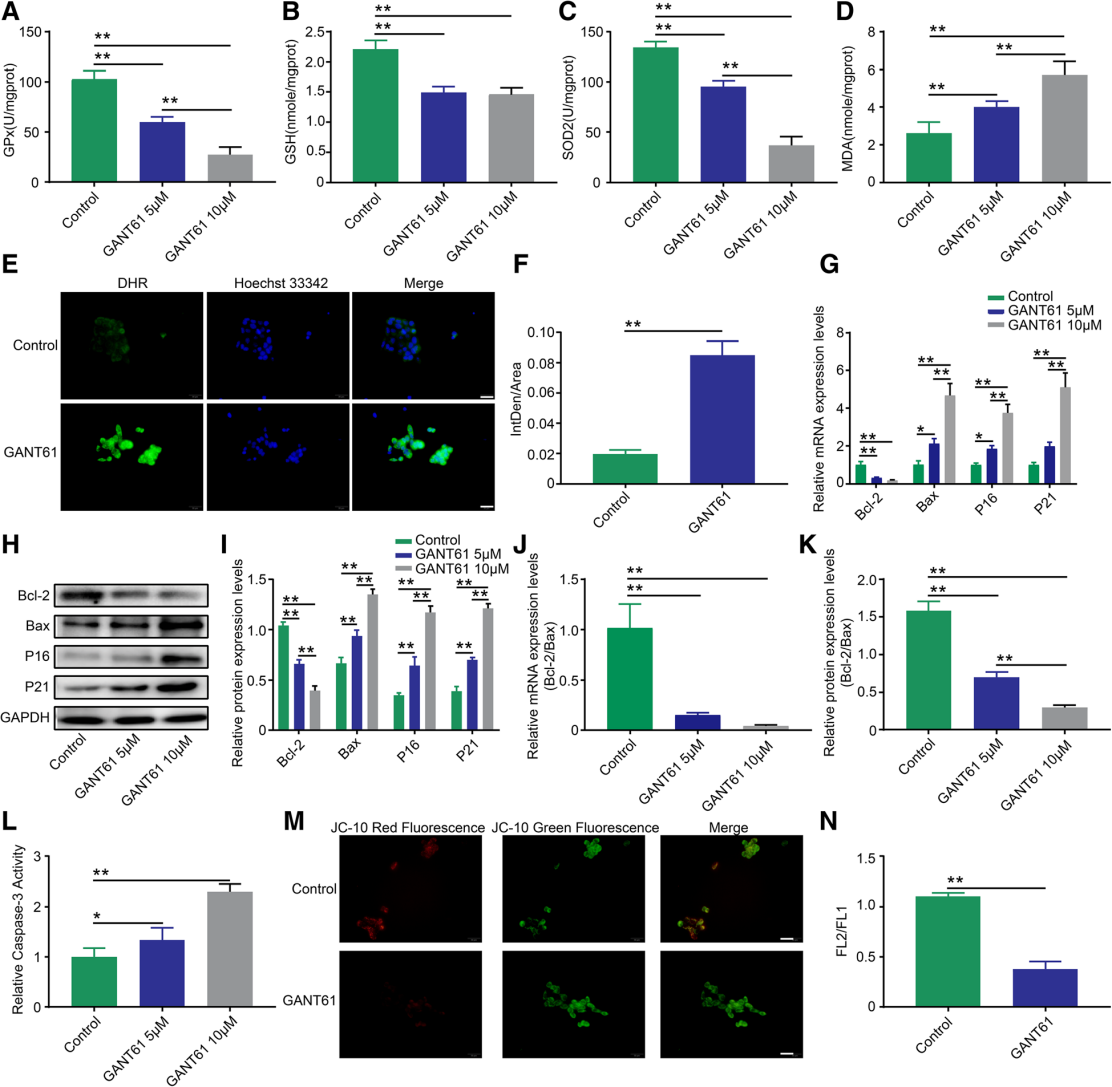
Suppressing Hh pathway activity reduced the antioxidative stress ability of FGSCs and induced apoptosis. Measurement of GPx (**a**) and SOD2 (**c**) enzyme activity. Levels of GSH (**b**) and MDA (**d**) in FGSCs. **e**, **f** Intracellular ROS levels detected by DHR, the scale is 20 μm. **g** mRNA expression levels of Bcl-2, Bax, P16, and P21. **h**, **i** Protein expression levels of Bcl-2, Bax, P16, and P21 in FGSCs. **j**, **k** mRNA and protein ratios of Bcl-2 to Bax expression levels. **l** Detection of Caspase-3 enzyme activity in FGSCs. **m**. **n** Change in mitochondrial membrane potential levels by JC-10 probe detection, the scale is 20 μm. *p < 0.05 and **p < 0.01

Stem cells are sensitive to their surroundings, and our results showed that the FGSC phenotype was influenced by the altered Hh pathway activity. Oxidative stress, defined as an imbalance between the production of reactive oxygen species (ROS) and antioxidants, is thought to contribute to cellular apoptosis or senescence. Therefore, to demonstrate whether FGSCs lost oxidative stress homeostasis in response to changes in Hh pathway activity, we administered GANT61 to FGSCs for 48 h and analyzed the expression of oxidative stress-associated molecules GPx, SOD2, GSH, and MDA. As shown in Fig. 6, a significant reduction in GPx (Fig. 6a, *p* < 0.01), GSH (Fig. 6b, *p* < 0.01), and SOD2 (Fig. 6c, *p* < 0.01) levels was evident in the 5 μM and 10 μM groups; however, MDA levels increased concurrently with an increase in GANT61 dosage (Fig. 6d, *p* < 0.01), suggesting that FGSCs had experienced oxidative stress and loss of stemness capacity. Representative images of DHR staining demonstrated a dramatic increase in staining in the 10 μM group, indicating reactive oxygen accumulation in those FGSCs (Fig. 6e, f, *p* < 0.01). Given that the FGSCs had experienced a loss in proliferation ability, we attempted to measure the levels of apoptosis in these cells. As expected, both mRNA and protein levels of the anti-apoptotic factor Bcl-2 were downregulated after treatment with GANT61, and levels of the pro-apoptotic factors Bax, P16, and P21 were upregulated (Fig. 6g–i, *p* < 0.01 or *p* < 0.05). The Bcl-2/Bax ratio data showed that the mRNA ratio decreased by almost 95% and the protein ratio dropped by 80% at 10 μM, indicating that FGSCs underwent apoptosis after blocking the Hh pathway (Fig. 6j, k, *p* < 0.01 or *p* < 0.05). Furthermore, we also measured the activity of Caspase 3 in FGSC cellular homogenate, and the results showed a small increase in the 5 μM group and an over double increase in the 10 μM group (Fig. 6l, *p* < 0.01 or *p* < 0.05). In addition, the mitochondrial potential test indicated that JC-10 staining decreased remarkably in the 10 μM group, suggesting that the transmembrane potential was disrupted and that FGSCs were in an apoptotic state (Fig. 6m, n, *p* < 0.01).

## Discussion

In general, normal mice can reach sexual maturity at 36 days after birth and lose reproductive capacity after 10 months of age. Hence, in our experiments, we chose 1-week-, 6-week-, and 48-week-old mice to correspond to infancy, adulthood, and senescence phases, respectively. Previous studies have suggested that the Hh signaling pathway plays a key role in regulating ovarian function [37, 38]. Liu et al. [11] demonstrated that female mice were infertile after double knockout of the Hh pathway ligands desert hedgehog (Dhh) and Indian hedgehog (Ihh) because of a lack of steroid product androgen. Wang et al. [39] demonstrated that sonic hedgehog (Shh) supplementation has beneficial effects on oocyte maturation, indicating a functional role for the Hh pathway in folliculogenesis. In view of this, we first measured the Hh pathway activity at different ages. Gli1, Ptch1, and Cyclin D1 are key molecules in the Hh pathway, and their expression levels represent the activity of the pathway. As shown in Fig. 1a–c, both mRNA and protein expression levels of the 3 key molecules dropped dramatically at 6 weeks of age, and the decrease was even more pronounced at 48 weeks compared with 1 week of age, indicating that Hh signaling activity is the highest at a young age and gradually decreases with age. IHC staining also showed that Hh pathway activity decreased following mouse reproductive senescence (Fig. 1d). Taken together, our results suggest that Hh signaling pathway activity gradually decreases with mouse reproductive age.

It has been proven that Hh pathway activity decreases with mouse age, but the correlation between these activities is still unknown. First, we measured the reproductive activity of normal mice at different ages. Compared with 1 week of age, both Mvh and Oct4 expression levels decreased at 6 weeks of age, even more so at 48 weeks of age (Fig. 2a–d). Therefore, our data suggest that Hh pathway activity corresponds to ovarian reproductive ability. To conceptualize the relevance, we selected the key molecule Gli1 to compare the coexpression with Mvh and Oct4 at the tissue level. The OSE is also known as the germinal epithelium, which is considered the main region containing germline cells. We attempted to detect whether there is coexpression of Gli1 and Mvh/Oct4 in the OSE. As shown in Fig. 2e and f, dual-IF revealed colocalization of Gli1 and Mvh/Oct4 in germinal epithelium, and the fluorescence intensity gradually decreased with aging, indicating a consistent correlation between the ovary reproductive capacity and Hh signaling activity in physiological conditions. Thus, our results strongly suggest that germline cell number or proliferation (including for FGSCs) has a close correlation with Hh signaling activity.

In addition to normal physiological mice, we attempted to assess the relationship between germline cells and the Hh pathway in a pathological mouse model. Treatment with cyclophosphamide (CTX) through intraperitoneal injection of 4- to 6-week-old mice was used to successfully generate a premature ovarian failure model [40]. HE staining revealed a decrease in normal follicles and an increase in atretic follicles in POF ovaries (Fig. 3a, b). In addition, no primary follicles and FGSCs were observed in the pathological OSE (Fig. 3c). Mvh and Oct4 expression levels exhibited an obvious decrease in the POF group. The above results confirmed that the number of germline cells or FGSCs decreased sharply in the POF model. Interestingly, the expression levels of Gli1, Ptch1, and Cyclin D1 also declined to varying degrees (Fig. 3d–f), indicating that Hh signaling activity decreases when the ovary is under pathological conditions. Therefore, the phenotype alteration in POF indicated that the Hh pathway regulates FGSC proliferation or stemness and follicle development.

To prove our hypothesis, the Hh pathway was blocked with inhibitor GANT61 to observe the phenotype of follicles, oocytes, and/or FGSCs in ovarian tissue. Given the convenience of ovary culture methods and follicle counting, we chose postnatal 3–5-day ovaries to study the effect of GANT61 in vitro. As expected, HE staining showed that a mass of primary follicles had disappeared in the GANT61 group, suggesting an occurrence of follicular atresia or damaged oocytes (Fig. 4a). Although FGSCs are mainly located in the OSE, they are difficult to distinguish by HE staining. Germline cells can specifically express the Mvh marker [41], stem cells are Oct4 positive [42, 43], and cells stained with both Mvh and Oct4 are considered FGSCs. To count the number of FGSCs, dual-IF was used to detect the number of FGSCs in ovarian sections. Our data showed that the number of FGSCs in the 10 μM group dropped over 50% compared with the DMSO group (Fig. 4b, e). Given that IHC cannot simultaneously detect the expression of Mvh and Oct4, we counted Mvh- and Oct4-positive cells individually. IHC staining showed that the number of Mvh-positive cells was over double that of FGSCs, indicating that the stained cells included oocytes or follicles as well as FGSCs (Fig. 4c, e). Because the number of Oct4-positive cells was greater than that of FGSCs, we speculated that there may be nonspecific staining of Oct4 (Fig. 4d, e). In addition, the mRNA and protein levels of Mvh and Oct4 were measured in the GANT61 group (Fig. 4f–h). As mentioned above, it is plausible that Hh pathway activity is involved in follicle development and FGSC proliferation at the tissue level. The inhibition of Hh signaling caused follicular atresia and FGSC depletion.

The above experiments proved that FGSC proliferation diminished when the Hh pathway was inhibited at the tissue level. However, whether Hh signaling also participates in regulating FGSC proliferation at the cell level remains unknown. As one of the newest verified stem cells, however, FGSCs are very difficult to isolate and culture in vitro because of their survival characteristics. In this study, we used a modified two-step enzymatic method to sort primary cells. In a previous method [44], ovaries were cut up as much as possible for the convenience of digestion. However, there is an obvious disadvantage to purifying FGSCs by this method because of contamination with other types of cells. Based on FGSCs mainly located in the OSE, in our experiment, instead of mincing the ovary, we cut an intact ovary into 2–4 sections, and the digestive time with collagenase IV and trypsin was carefully controlled to prevent over digestion. Using this method, FGSCs can be isolated at a maximum number and purification, and many FGSCs can be collected by centrifugation after removing the tissue masses. Finally, we successfully obtained two different types of cells that expressed the stemness markers Oct4, Stella, Fragilis, and Nanog [45, 46] and suggested pluripotent properties (Additional file 1: Figure S1A and B). In addition, ALP staining and double IF further verified that the isolated cells were FGSCs (Additional file 1: Figure S1C, D, and E). One of the cell types exhibited a beaded growth pattern and a slow proliferation speed, and the other multiplied quickly and showed a colony-like growth model. Considering the cell proliferation rate and maintenance of activity in vitro, we chose the colony-like FGSCs to perform further experiments. To assess the function of Hh signaling in the regulation of FGSC proliferation in vitro, colony-like FGSCs were subcultured and treated with 5 μM or 10 μM GANT61. As shown in Fig. 5a–c, the FGSCs lost their growth capacity or viability and stemness after inhibition of Hh pathway activity. Proliferating cells can be identified through the DNA replication process, so EdU staining can be used to count proliferating FGSCs. To better show the results, we combined Mvh or Oct4 with EdU by dual-IF staining. Our experimental data proved that blocking Hh signaling contributes to the inhibition of DNA replication and leads to a decrease in the proliferative capacity of FGSCs (Fig. 5d–f). We also measured the expression levels of Mvh and Oct4 in vitro. The results showed that both Mvh and Oct4 levels were downregulated in GANT61 groups (Fig. 5g–i), and dual-IF staining confirmed these results (Fig. 5j, k). Taken together, our results suggest that inhibition of Hh signaling inhibited FGSC proliferation or growth in vitro and reduced the reproductive capacity and stemness of FGSCs.

The phenotype of stem cells is affected by the homeostasis of their microenvironment [47–49]. In the *Drosophila* ovary, Lu et al. [38] discovered that Hh signaling participates in regulating the function of the germline stem cell progeny differentiation niche. For FGSCs, whether the cells are influenced by their microenvironment or niche via changes in the Hh pathway remains poorly understood. Abnormally high oxidative stress can generate a large number of oxidative intermediate products, such as ROS [50], which destroys the homeostasis of the local microenvironment and damages the normal functions of cells. Some evidence has shown that excessive ROS accumulation can inhibit Shh expression by blocking the PI3K/AKT pathway and restricting downstream GSK-3β phosphorylation [51]. Song et al. [52] discovered that the upregulation of Gli1 reduced the accumulation of intracellular ROS production induced by high glucose concentrations. In addition, Hai et al. [53] also proved that the Shh ligand has an antagonistic effect on oxidative stress injury via the PI3K/AKT/ Bcl-2 pathway, suggesting that the Hh pathway may regulate the survival fate of stem cells through oxidative stress-related mechanisms. The accumulation of ROS depends on the ratio between the oxidant and the antioxidant system, and antioxidant enzymes such as GPx and SOD2 can effectively clear ROS in the cell [54, 55]. Therefore, we measured the enzymatic activities of GPx and SOD2 and the content changes of the correlative molecules GSH and MDA. The results showed that the intracellular GPx and SOD2 enzyme activities were inhibited significantly in FGSCs after GANT61 treatment; the results also showed a GSH decrease and an MDA increase (Fig. 6a–d), indicating that the blockage of Hh signaling damaged FGSCs through activating oxidative stress [56, 57]. In addition, DHR, an examination index of ROS that presents green fluorescence when it is oxidized in mitochondria, was obviously enhanced as shown by an increase in green fluorescence in the GANT61 group (Fig. 6e, f), indicating a large amount of ROS products in FGSCs. Previous studies have shown that the accumulation of ROS can damage the mitochondria and trigger apoptosis [58, 59]. The Bcl-2 family mainly includes the anti-apoptotic protein Bcl-2 and the pro-apoptotic molecule Bax, and their balance maintains normal programmed apoptosis [60, 61]. Nye et al. [62] demonstrated that Gli1, as an effector of TGF-β signaling, can induce Bcl-2 expression via direct binding to the Bcl-2 promoter. PCAF, a histone acetyltransferase, mediates the Gli1 versus Bcl-2/Bax axis by acetylating Gli1 and preventing its entry into the nucleus to regulate the induction of apoptosis [63]. In our experiments, the expression levels of Bcl-2 and Bax varied, and dramatic upregulation also occurred in the apoptosis marker molecules P16, P21, and Caspase 3 (Fig. 6g–i, l), indicating that the Hh pathway participated in the apoptosis pathway in FGSCs. The ratio of Bcl-2 to Bax can be used to evaluate the degree of apoptosis, as shown in Fig. 6j and k. Both the mRNA and protein ratio dropped dramatically in the GANT61 group, demonstrating that blocking the Hh pathway triggered the apoptosis in FGSCs. The mitochondria are closely related to apoptosis, and the disrupted transmembrane potential is identified as a marker for apoptosis [64, 65]. JC-10, a fluorescent dye used as an indicator of transmembrane potential, can change its color from green to orange due to the increase of potential [66]. As depicted in Fig. 6m and n, red fluorescence was obviously decreased in the GANT61 group, suggesting a loss of potential and a possible occurrence of apoptosis. Overall, our data confirmed that blocking the Hh pathway disrupted oxidative stress homeostasis and caused ROS accumulation and apoptosis in FGSCs, which contributed to the loss of FGSC stemness and proliferation.

Compared to the prolongation of life expectancy through the development of life science exploration and clinical therapeutic techniques, the female reproductive age has not increased. As a newly discovered stem cell type, FGSCs can differentiate into oocytes and prompt follicular renewal [67]. Therefore, a question remains why the pregnancy age is still limited even though FGSCs have the capacity of oocyte regeneration. One possible explanation is that the proliferation or stemness of FGSCs is controlled by the microenvironment or niche so that the number of FGSCs is not enough to make up for oocyte consumption. Our data revealed that the Hh pathway plays an important role in the regulation of follicle development and FGSC proliferation via the alteration of antioxidative stress ability and apoptosis. In POF or aging ovaries, the reduction of Hh activity disrupts the supporting niche of the follicles or FGSCs and induces follicle atresia and FGSC apoptosis. Therefore, a suitable regulation of Hh pathway activity can relieve oxidative stress, improve mitochondrial function, and protect against apoptosis, which may provide a new treatment method or strategy for clinically infertile patients. Our research is also beneficial to deepening our understanding of the endocrine function of the ovary and hormone levels for ovarian dysfunction as related to the Hh pathway, which regulates folliculogenesis and FGSC reproduction. In addition, treatment with chemotherapy drugs that affect the ovaries inevitably leads to excessive ROS accumulation and oxidative stress. Our other ongoing study discovered that resveratrol (Res) can clear ROS accumulation and relieve oxidative stress via regulating the Hh pathway (our unpublished data). Hence, our findings present new prospects for promoting oocyte regeneration, delaying menopause, and restoring ovarian function.

At present, studies on FGSCs in postnatal mammals are seldom reported, mainly due to the difficulty of isolating, subculturing, and maintaining FGSCs in vitro. Although our lab has tried to optimize the culture medium and method, the survival and subculture of FGSCs need to be further improved. In this study, we focused on inhibiting the Hh signaling pathway to regulate follicle development and FGSC proliferation, which has certain limitations. Next, we plan to explore the rescue of ovarian function in senescent or POF mice by restoring normal follicles and prompting FGSC differentiation via activating the Hh pathway.

## Conclusion

Taken together, although further study is clearly required to elucidate the mechanism between the Hh pathway and follicular development and FGSC proliferation, our data established that the Hh pathway, a canonical signaling pathway in connection with cell proliferation and tissue polarity, is a crucial regulator of follicle development and FGSC reproduction. Our findings suggest that inhibiting Hh signaling activity directly contributes to follicular atresia and reduction in FGSC reproductive capacity or stemness, providing a new regulatory mechanism for FGSC proliferation.

## Additional file

Additional file 1: A: microscopic observation of C-FGSCs (colony-like FGSCs) and B-FGSCs (beaded-like FGSCs), the scale is 20 μm; B: DNA agarose electrophoresis of multi-stemness molecular markers; C: ALP staining of FGSCs, scale is 20 μm; D: double IF of Mvh and Oct4, scale is 20 μm; E: double IF of Mvh and EdU, the scale is 20 μm. (DOCX 320 kb)

## Data Availability

The datasets used and analyzed during the current study are available from the corresponding author on reasonable request.

## Abbreviations

ALP: Alkaline phosphatase
Dhh: Desert hedgehog
DHR: Dihydrorhodamine
FGSCs: Female germline stem cells
GPx: Glutathione peroxidase
GSH: Glutathione
Hh: Hedgehog
Ihh: Indian hedgehog
MDA: Malondialdehyde
Mvh: Mouse Vasa homolog
Oct4: Pou5f1, POU domain, class 5, transcription factor 1
OGSCs: Ovarian germline stem cells
OSE: Ovarian surface epithelium
POF: Premature ovarian failure
POI: Primary ovarian insufficiency
ROS: Reactive oxygen species
Shh: Sonic hedgehog
SOD2: Superoxide dismutase 2

## References

[1] Bhartiya D (2015). Ovarian stem cells are always accompanied by very small embryonic-like stem cells in adult mammalian ovary. J Ovarian Res..

[2] Virant-Klun I, Skutella T, Stimpfel M (2011). Ovarian surface epithelium in patients with severe ovarian infertility: a potential source of cells expressing markers of pluripotent/multipotent stem cells. J Biomed Biotechnol.

[3] Virant-Klun I, Zech N, Rozman P (2008). Putative stem cells with an embryonic character isolated from the ovarian surface epithelium of women with no naturally present follicles and oocytes. Differentiation..

[4] Zou K, Hou L, Sun K (2011). Improved efficiency of female germline stem cell purification using fragilis-based magnetic bead sorting. Stem Cells Dev.

[5] Zou K, Yuan Z, Yang Z (2009). Production of offspring from a germline stem cell line derived from neonatal ovaries. Nat Cell Biol.

[6] Bhartiya D, Sriraman K, Parte S (2013). Ovarian stem cells: absence of evidence is not evidence of absence. J Ovarian Res.

[7] Esmaeilian Y, Atalay A, Erdemli E (2017). Putative germline and pluripotent stem cells in adult mouse ovary and their in vitro differentiation potential into oocyte-like and somatic cells. Zygote..

[8] Regan JL, Schumacher D, Staudte S (2017). Non-canonical hedgehog signaling is a positive regulator of the WNT pathway and is required for the survival of colon cancer stem cells. Cell Rep.

[9] Zhang M, Lin YH, Sun YJ (2016). Pharmacological reprogramming of fibroblasts into neural stem cells by signaling-directed transcriptional activation. Cell Stem Cell.

[10] Chen H, Zuo Q, Wang Y (2017). Regulation of hedgehog signaling in chicken embryonic stem cells differentiation into male germ cells (Gallus). J Cell Biochem.

[11] Liu C, Peng J, Matzuk MM, et al. Lineage specification of ovarian theca cells requires multicellular interactions via oocyte and granulosa cells. Nat Commun. 2015;6. 10.1038/ncomms7934 PMC4413950 25917826

[12] Hsu TH, Yang CY, Yeh TH (2017). The hippo pathway acts downstream of the hedgehog signaling to regulate follicle stem cell maintenance in the Drosophila ovary. Sci Rep.

[13] Roberts KJ, Kershner AM, Beachy PA (2017). The stromal niche for epithelial stem cells: a template for regeneration and a brake on malignancy. Cancer Cell.

[14] Guan S, Xie L, Ma T, et al. Effects of melatonin on early pregnancy in mouse: involving the regulation of StAR, Cyp11a1, and Ihh expression. Int J Mol Sci. 2017;18. 10.3390/ijms18081637 PMC5578027 28749439

[15] Song K, Ma W, Huang C (2016). Expression pattern of mouse vasa homologue (MVH) in the ovaries of C57BL/6 female mice. Med Sci Monit.

[16] Kobayashi T, Kajiura-Kobayashi H, Nagahama Y (2000). Differential expression of vasa homologue gene in the germ cells during oogenesis and spermatogenesis in a teleost fish, tilapia, Oreochromis niloticus. Mech Dev.

[17] Noce T, Okamoto-Ito S, Tsunekawa N (2001). Vasa homolog genes in mammalian germ cell development. Cell Struct Funct.

[18] Pesce M, Scholer HR (2000). Oct-4: control of totipotency and germline determination. Mol Reprod Dev.

[19] Ma X, Li P, Sun X (2018). Differentiation of female Oct4-GFP embryonic stem cells into germ lineage cells. Cell Biol Int.

[20] Eguizabal C, Shovlin TC, Durcova-Hills G (2009). Generation of primordial germ cells from pluripotent stem cells. Differentiation..

[21] Didiasova Miroslava, Schaefer Liliana, Wygrecka Malgorzata (2018). Targeting GLI Transcription Factors in Cancer. Molecules.

[22] Choi CH, Ryu JY, Cho YJ (2017). The anti-cancer effects of itraconazole in epithelial ovarian cancer. Sci Rep.

[23] Lin Z, Sheng H, You C (2017). Inhibition of the CyclinD1 promoter in response to sonic hedgehog signaling pathway transduction is mediated by Gli1. Exp Ther Med..

[24] Kurebayashi J, Koike Y, Ohta Y (2017). Anti-cancer stem cell activity of a hedgehog inhibitor GANT61 in estrogen receptor-positive breast cancer cells. Cancer Sci.

[25] Gonnissen Annelies, Isebaert Sofie, McKee Chad, Muschel Ruth, Haustermans Karin (2017). The Effect of Metformin and GANT61 Combinations on the Radiosensitivity of Prostate Cancer Cells. International Journal of Molecular Sciences.

[26] Jung CH, Kim EM, Song JY (2019). Mitochondrial superoxide dismutase 2 mediates gamma-irradiation-induced cancer cell invasion. Exp Mol Med.

[27] Perez-Torres I, Torres-Narvaez JC, Guarner-Lans V (2019). Myocardial protection from ischemia-reperfusion damage by the antioxidant effect of Hibiscus sabdariffa Linnaeus on metabolic syndrome rats. Oxidative Med Cell Longev.

[28] Fang W, Chi Z, Li W (2019). Comparative study on the toxic mechanisms of medical nanosilver and silver ions on the antioxidant system of erythrocytes: from the aspects of antioxidant enzyme activities and molecular interaction mechanisms. J Nanobiotechnology.

[29] Chen X, Wang L, Hou J (2019). Study on the dynamic biological characteristics of human bone marrow mesenchymal stem cell senescence. Stem Cells Int.

[30] Boonekamp Jelle J., Bauch Christina, Mulder Ellis, Verhulst Simon (2017). Does oxidative stress shorten telomeres?. Biology Letters.

[31] Du J, Cai J, Wang S (2017). Oxidative stress and apotosis to zebrafish (Danio rerio) embryos exposed to perfluorooctane sulfonate (PFOS) and ZnO nanoparticles. Int J Occup Med Environ Health.

[32] Maes ME, Schlamp CL, Nickells RW (2017). BAX to basics: how the BCL2 gene family controls the death of retinal ganglion cells. Prog Retin Eye Res.

[33] Sadek A, Sheneef A, Sabet EA (2017). The role of Bcl-2 and Bax as markers of disease progression in hepatitis C virus infected patients. Egypt J Immunol.

[34] Liu C, Vojnovic D, Kochevar IE (2016). UV-A irradiation activates Nrf2-regulated antioxidant defense and induces p53/Caspase3-dependent apoptosis in corneal endothelial cells. Invest Ophthalmol Vis Sci.

[35] Chiu CH, Chou YC, Lin JP (2015). Chloroform extract of Solanum lyratum induced G0/G1 arrest via p21/p16 and induced apoptosis via reactive oxygen species, caspases and mitochondrial pathways in human oral cancer cell lines. Am J Chin Med.

[36] Zhang JX, Han YP, Bai C (2015). Notch1/3 and p53/p21 are a potential therapeutic target for APS-induced apoptosis in non-small cell lung carcinoma cell lines. Int J Clin Exp Med.

[37] Richards JS, Ren YA, Candelaria N (2018). Ovarian follicular Theca cell recruitment, differentiation, and impact on fertility: 2017 update. Endocr Rev.

[38] Lu T, Wang S, Gao Y (2015). COP9-Hedgehog axis regulates the function of the germline stem cell progeny differentiation niche in the Drosophila ovary. Development..

[39] Wang DC, Huang JC, Lo NW (2017). Sonic Hedgehog promotes in vitro oocyte maturation and term development of embryos in Taiwan native goats. Theriogenology..

[40] Zhang Q, Xu M, Yao X (2015). Human amniotic epithelial cells inhibit granulosa cell apoptosis induced by chemotherapy and restore the fertility. Stem Cell Res Ther.

[41] Afsartala Z, Rezvanfar MA, Hodjat M (2016). Amniotic membrane mesenchymal stem cells can differentiate into germ cells in vitro. In Vitro Cell Dev Biol Anim.

[42] Picot T, Aanei CM, Fayard A (2017). Expression of embryonic stem cell markers in acute myeloid leukemia. Tumour Biol.

[43] Reeve RL, Yammine SZ, Morshead CM (2017). Quiescent Oct4+ neural stem cells (NSCs) repopulate ablated glial fibrillary acidic protein+ NSCs in the adult mouse brain. Stem Cells.

[44] Wu M, Xiong J, Ma L (2018). Enrichment of female germline stem cells from mouse ovaries using the differential adhesion method. Cell Physiol Biochem.

[45] Zou K, Wang J, Bi H (2019). Comparison of different in vitro differentiation conditions for murine female germline stem cells. Cell Prolif.

[46] Adib S, Valojerdi MR (2017). Molecular assessment, characterization, and differentiation of theca stem cells imply the presence of mesenchymal and pluripotent stem cells in sheep ovarian theca layer. Res Vet Sci.

[47] Gattazzo F, Urciuolo A, Bonaldo P (2014). Extracellular matrix: a dynamic microenvironment for stem cell niche. Biochim Biophys Acta.

[48] Sanchez-Aguilera A, Mendez-Ferrer S (2017). The hematopoietic stem-cell niche in health and leukemia. Cell Mol Life Sci.

[49] Sailaja BS, He XC, Li L (2016). The regulatory niche of intestinal stem cells. J Physiol.

[50] Denu RA, Hematti P (2016). Effects of oxidative stress on mesenchymal stem cell biology. Oxidative Med Cell Longev.

[51] Zhao W, Pan X, Li T (2016). Lycium barbarum polysaccharides protect against trimethyltin chloride-induced apoptosis via sonic hedgehog and PI3K/Akt signaling pathways in mouse neuro-2a cells. Oxidative Med Cell Longev.

[52] Song N, Wang H, Gu T (2018). Sonic hedgehog-c-Jun N-terminal kinase-zinc finger protein Gli1 signaling protects against high glucose concentration-induced reactive oxygen species generation in human fibroblasts. Exp Ther Med.

[53] Hai B, Zhao Q, Deveau MA (2018). Delivery of sonic hedgehog gene repressed irradiation-induced cellular senescence in salivary glands by promoting DNA repair and reducing oxidative stress. Theranostics..

[54] Quan M, Cai CL, Valencia GB (2017). MnTBAP or catalase is more protective against oxidative stress in human retinal endothelial cells exposed to intermittent hypoxia than their co-administration (EUK-134). React Oxyg Species (Apex).

[55] Cheng Y, Dai C, Zhang J (2017). SIRT3-SOD2-ROS pathway is involved in linalool-induced glioma cell apoptotic death. Acta Biochim Pol.

[56] Prasad DKV, Satyanarayana U, Shaheen U (2017). Oxidative stress in the development of genetic generalised epilepsy: an observational study in southern Indian population. J Clin Diagn Res.

[57] Alghobashy AA, Alkholy UM, Talat MA (2018). Trace elements and oxidative stress in children with type 1 diabetes mellitus. Diabetes Metab Syndr Obes.

[58] Liang J, Cao R, Wang X (2017). Mitochondrial PKM2 regulates oxidative stress-induced apoptosis by stabilizing Bcl2. Cell Res.

[59] Zhang L, Fang Y, Xu XF (2017). Moscatilin induces apoptosis of pancreatic cancer cells via reactive oxygen species and the JNK/SAPK pathway. Mol Med Rep.

[60] Gross A (2016). BCL-2 family proteins as regulators of mitochondria metabolism. Biochim Biophys Acta.

[61] Kourtis A, Adamopoulos PG, Papalois A (2018). Quantitative analysis and study of the mRNA expression levels of apoptotic genes BCL2, BAX and BCL2L12 in the articular cartilage of an animal model of osteoarthritis. Ann Transl Med.

[62] Nye MD, Almada LL, Fernandez-Barrena MG (2014). The transcription factor GLI1 interacts with SMAD proteins to modulate transforming growth factor beta-induced gene expression in a p300/CREB-binding protein-associated factor (PCAF)-dependent manner. J Biol Chem.

[63] Gai X, Tu K, Li C (2015). Histone acetyltransferase PCAF accelerates apoptosis by repressing a GLI1/BCL2/BAX axis in hepatocellular carcinoma. Cell Death Dis.

[64] Barnwal B, Karlberg H, Mirazimi A (2016). The non-structural protein of Crimean-Congo hemorrhagic fever virus disrupts the mitochondrial membrane potential and induces apoptosis. J Biol Chem.

[65] Zhang HL, Zhang H (2017). Withaferin-a induces apoptosis in osteosarcoma U2OS cell line via generation of ROS and disruption of mitochondrial membrane potential. Pharmacogn Mag.

[66] Patel H, Chen J, Das KC (2013). Hyperglycemia induces differential change in oxidative stress at gene expression and functional levels in HUVEC and HMVEC. Cardiovasc Diabetol.

[67] Wu C, Xu B, Li X (2017). Tracing and characterizing the development of transplanted female germline stem cells in vivo. Mol Ther.

