# Supplementary material for: Hedgehog pathway inhibition causes primary follicle atresia and decreases female germline stem cell proliferation capacity or stemness
Source: Stem Cell Res Ther. 2019 Jul 5;10:198. doi: 10.1186/s13287-019-1299-5 (PMC6612207; doi:10.1186/s13287-019-1299-5)
Supplement: Supplementary file 1 — A: microscopic observation of C-FGSCs (colony-like FGSCs) and B-FGSCs (beaded-like FGSCs), the scale is 20 μm; B: DNA agarose electrophoresis of multi-stemness molecular markers; C: ALP staining of FGSCs, scale is 20 μm; D: double IF of Mvh and Oct4, scale is 20 μm; E: double IF of Mvh and EdU, the scale is 20 μm. (DOCX 320 kb) [file 13287_2019_1299_MOESM1_ESM.docx]

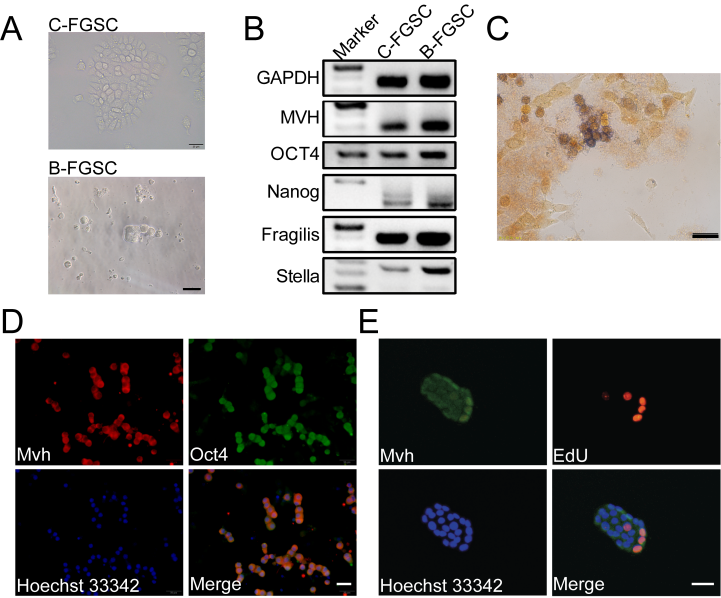


Additional file 1 A: Microscopic observation of C-FGSCs (Colony-like FGSCs) and B-FGSCs (Beaded-like FGSCs), the scale is 20 μm; B: DNA agarose electrophoresis of multi-stemness molecular markers; C: ALP staining of FGSCs, scale is 20 μm; D: Double IF of Mvh and Oct4, scale is 20 μm; E: Double IF of Mvh and EdU, the scale is 20 μm.
